# Recurrent Hyperammonemic Encephalopathy in Adults with Citrin Deficiency: A Case Report of Two Genetically Confirmed Cases

**DOI:** 10.3390/jcm15176741

**Published:** 2026-08-30

**Authors:** Tram Nguyen Que Pham, Van Huy Vo, Qui Huu Nguyen, Thuy Thi Thanh Trinh, Thong Duy Vo

**Affiliations:** 1 Department of Gastroenterology, University Medical Center Ho Chi Minh City, Ho Chi Minh City 72714, Vietnam; 2 Department of Internal Medicine, School of Medicine, University of Medicine and Pharmacy at Ho Chi Minh City, Ho Chi Minh City 72714, Vietnam

**Keywords:** citrin deficiency, *SLC25A13*, hyperammonemia, metabolic encephalopathy, case report

## Abstract

**Background**: Adolescent and adult citrin deficiency (AACD), formerly known as adult-onset citrullinemia type II, is a rare autosomal recessive disorder caused by biallelic pathogenic variants in *SLC25A13*. It is an underrecognized cause of recurrent hyperammonemic encephalopathy, particularly when hepatic function is relatively preserved. **Methods**: We report two unrelated Vietnamese young men, aged 21 and 18 years, who presented with recurrent neuropsychiatric episodes. Clinical, biochemical, imaging, electrophysiological, and genetic findings were evaluated; whole-exome sequencing findings were confirmed by Sanger sequencing. **Results**: Both patients had long-standing preferences for protein- and fat-rich foods and avoidance of carbohydrate-rich foods, together with episodic hyperammonemia (345.21 and 103.07 µmol/L during symptomatic episodes). The first patient had a history of neonatal jaundice, mild cirrhosis, and severe behavioral disturbances, whereas the second was markedly lean and had no structural liver disease. Acquired causes of hyperammonemia and portosystemic shunting were excluded. Both patients harbored the homozygous pathogenic *SLC25A13* variant NM_014251.3.852_855del (p.Met285ProfsTer2). Ammonia-lowering therapy and a low-carbohydrate, protein- and fat-enriched diet supplemented with medium-chain triglycerides resulted in clinical improvement, with no recurrent encephalopathic episodes during 6 months of follow-up in either patient. **Conclusions**: AACD should be considered in adolescents and adults with otherwise unexplained recurrent hyperammonemic encephalopathy, especially when ammonia elevation is disproportionate to liver disease. Characteristic dietary preferences provide an important diagnostic clue, and molecular testing enables definitive diagnosis and timely management.

## 1. Introduction

Acute hyperammonemic encephalopathy in adults is most commonly associated with advanced liver disease or portosystemic shunting. However, a subset of patients develop recurrent neuropsychiatric manifestations despite relatively preserved hepatic function, suggesting an underlying inherited metabolic disorder [1]. Among these, citrin deficiency is an important but frequently overlooked cause because its clinical presentation often mimics hepatic encephalopathy, epilepsy, intracranial disease, or primary psychiatric disorders [2,3].

Citrin deficiency is an autosomal recessive disorder caused by biallelic pathogenic variants in *SLC25A13*, located on chromosome 7q21.3 [4]. The gene encodes citrin, a calcium-dependent mitochondrial aspartate–glutamate carrier expressed predominantly in the liver [5]. Citrin is an essential component of the malate–aspartate shuttle and facilitates the export of mitochondrial aspartate to the cytosol, thereby supporting urea-cycle function, nucleotide synthesis, glycolysis, and cellular redox homeostasis. Loss of citrin function impairs hepatic energy metabolism and limits the availability of cytosolic aspartate for ammonia detoxification [6].

More than 100 pathogenic *SLC25A13* variants have been reported, including frameshift, splice-site, nonsense, missense, insertion, duplication, and large deletion variants. Their distribution differs substantially across populations [7]. The recurrent four-base-pair deletion NM_014251.3:c.852_855del (p.Met285ProfsTer2), historically reported as 851del4 or c.851_854del in earlier publications, is one of the most frequently reported pathogenic variants in East Asian populations [3,7]. In a Vietnamese pediatric cohort of 292 unrelated patients with neonatal intrahepatic cholestasis caused by citrin deficiency, the corresponding recurrent deletion accounted for 91.78% of identified mutant alleles [8]. However, these data were derived primarily from pediatric NICCD cases and should not be interpreted as the variant frequency specifically among Vietnamese adults with AACD.

AACD is characterized by recurrent episodes of hyperammonemic encephalopathy with diverse neuropsychiatric manifestations, often accompanied by a characteristic preference for protein- and fat-rich foods and avoidance of carbohydrates [9]. Early recognition is crucial because appropriate dietary intervention, medium-chain triglyceride (MCT) supplementation, and ammonia-lowering therapy can effectively prevent recurrent metabolic decompensation, whereas liver transplantation remains the only definitive treatment for severe disease [9,10]. Published molecular data on citrin deficiency in Vietnam have largely focused on pediatric patients with NICCD [8]. Although these studies have established a substantial burden of pathogenic *SLC25A13* variants in Vietnamese children, genetically confirmed adolescent and adult cases presenting with recurrent hyperammonemic encephalopathy remain sparsely documented. We therefore report two Vietnamese patients with genetically confirmed AACD who presented with recurrent hyperammonemic encephalopathy but showed distinct hepatic and neuropsychiatric phenotypes.

## 2. Case Reports

### 2.1. Case 1

A 21-year-old male presented with recurrent episodes of abnormal behavior and altered consciousness over the past three months. The first event involved repetitive wandering and perseverative speech lasting about one hour, followed by full recovery without recollection. A month later, he experienced a longer episode lasting about six hours and was evaluated at another hospital, where a possible brain lesion was suspected and citicoline was prescribed. Subsequent episodes became more severe, characterized by agitation, disinhibition, inappropriate undressing, and shouting, without convulsions or incontinence. His history included neonatal jaundice and small stature since childhood. Long before the onset of neuropsychiatric symptoms, he had consistently preferred protein-rich foods and had avoided rice, sweets, and other carbohydrate-rich foods. Over the preceding six months, nocturnal restlessness and incoherent speech had become frequent, with slower responsiveness during the day. A maternal uncle had a similar adult-onset behavioral disorder previously diagnosed as epilepsy. No other family member was known to have recurrent encephalopathy, unexplained hyperammonemia, neonatal cholestasis, or a similarly marked aversion to carbohydrate-rich foods.

**Figure 1:**
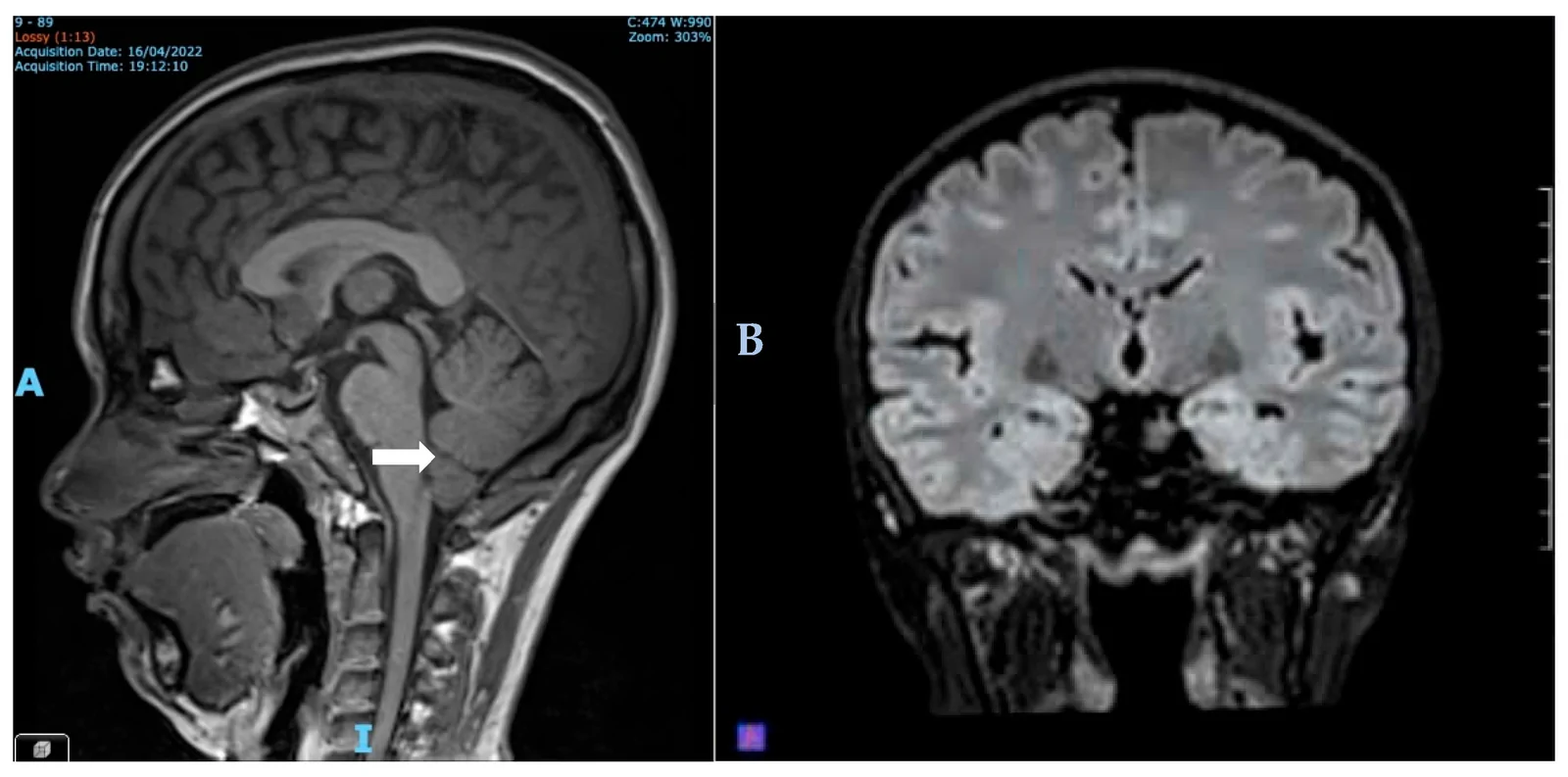
Brain MRI showing (**A**) low-lying cerebellar tonsils below the foramen magnum (white arrow) in Case 1, and (**B**) normal findings in Case 2.

**Figure 2:**
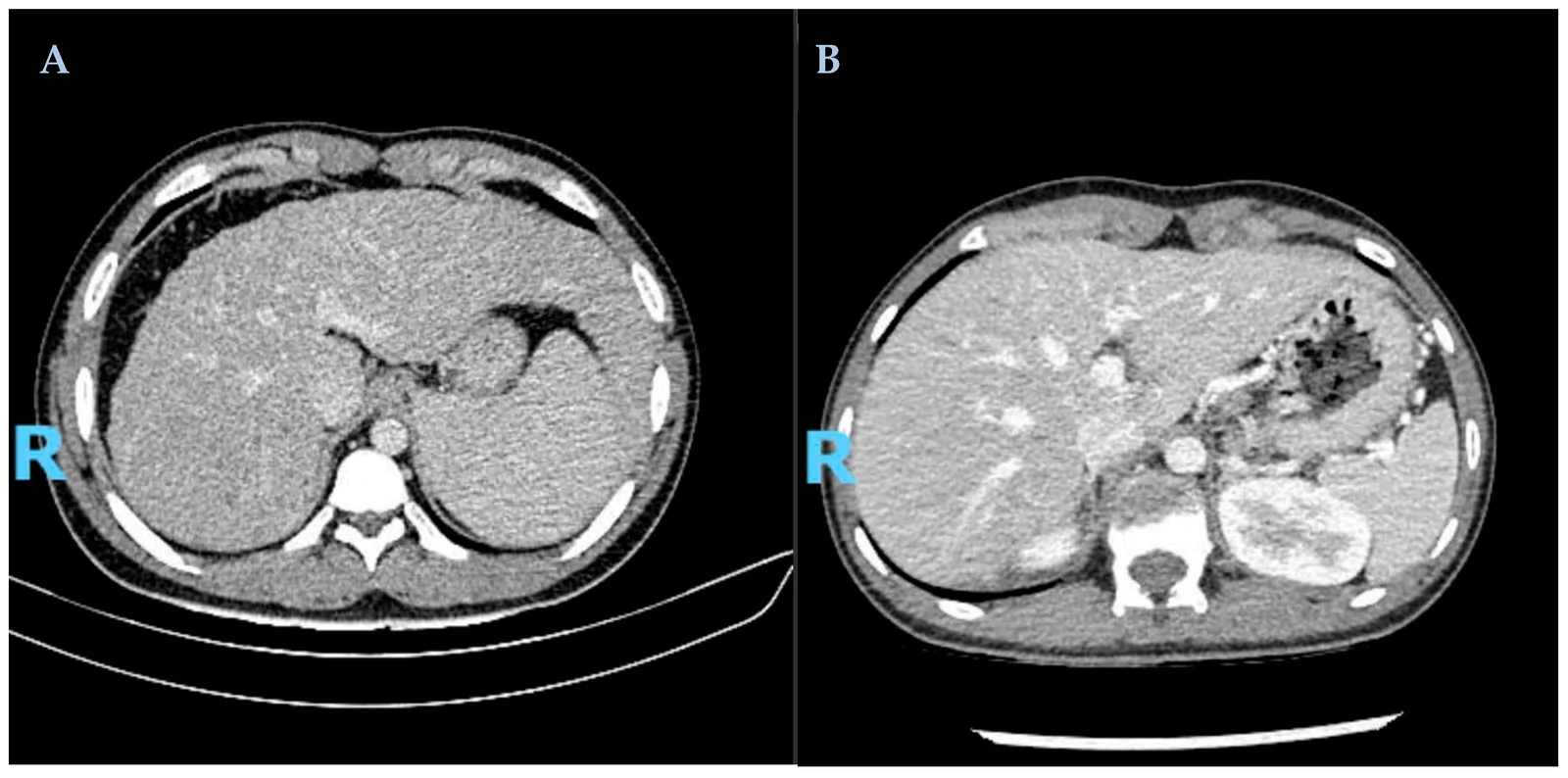
Abdominal CT showing (**A**) cirrhosis in Case 1 and (**B**) normal liver in Case 2.

On admission, the patient was alert and oriented, with normal speech and cognition. Cranial nerves were normal, muscle strength was full, and reflexes were brisk with bilateral Hoffman’s sign. Sensory, cerebellar, and meningeal signs were absent. However, on the following day, he developed progressive disorientation and incoherent speech, followed by agitation and shouting episodes. Examination revealed somnolence with impaired orientation to time and place, consistent with acute encephalopathy. At that time, he had not yet received any ammonia-lowering therapy. Further laboratory and imaging evaluations were performed to identify the underlying cause. Laboratory evaluation revealed mildly elevated liver enzymes with normal total bilirubin and albumin. Platelet count was slightly reduced, and INR was 1.22. Notably, plasma ammonia was markedly elevated at 345.21 µmol/L, indicating significant hyperammonemia. Abdominal CT demonstrated morphological features suggestive of compensated cirrhosis with recanalization of the umbilical vein and no portosystemic shunt. Representative MRI and CT findings are shown in Figure 1A and Figure 2A, respectively.

Because of the low ceruloplasmin level and altered mental status, Wilson’s disease was initially suspected; however, slit-lamp examination revealed no Kayser–Fleischer rings, and 24-h urinary copper excretion was normal. Viral markers for hepatitis B, hepatitis C, CMV, EBV, and HSV, as well as autoimmune serologies, were all negative. Brain MRI showed a small enhancing lesion beneath the left occipital cortex with subtle adjacent meningeal enhancement, as well as low-lying cerebellar tonsils approximately 7 mm below the foramen magnum. However, the patient had no fever, headache, focal neurological deficit, or meningeal signs, and cerebrospinal fluid analysis was normal. Therefore, the occipital lesion and meningeal enhancement were considered nonspecific and unlikely to account for the recurrent neuropsychiatric episodes. His symptoms resolved rapidly after ammonia-lowering therapy without antimicrobial, corticosteroid, antiepileptic, neurosurgical, or other lesion-directed treatment. No follow-up brain MRI was available; therefore, radiological resolution of these findings could not be assessed. EEG revealed diffuse slow-wave activity without epileptiform discharges, supporting metabolic encephalopathy.

Based on the marked hyperammonemia, ammonia-lowering therapy with L-ornithine–L-aspartate (LOLA), rifaximin, and lactulose was initiated, resulting in gradual improvement of consciousness. By the next day, he was fully alert, oriented, and exhibited fluent, coherent speech without further behavioral disturbances. Plasma ammonia decreased from 345 to 110 µmol/L following treatment.

Because the degree of hyperammonemia was out of proportion to the mild liver dysfunction, a metabolic cause was suspected. Genetic testing later revealed a homozygous c.852_855del (p.Met285ProfsTer2) mutation in the *SLC25A13* gene, consistent with AACD. After discharge, the patient continued treatment with lactulose, rifaximin, and LOLA, along with a low-carbohydrate, protein- and fat-enriched diet supplemented with MCT. During 6 months of follow-up after discharge, his clinical condition remained stable, and no further episodes of altered consciousness or behavioral disturbance were observed.

### 2.2. Case 2

The second patient was admitted approximately 3 months after the first patient. The two patients were not related, came from different families, and had no identified epidemiological relationship. An 18-year-old male had several brief episodes of confusion and abnormal behavior over the past year. Each episode began with sudden confusion, disinhibition, and drowsiness lasting for several hours, followed by complete recovery. These attacks occurred four to five times, often triggered by fatigue or large meals. During the most recent attack, his family noticed restlessness and purposeless behavior, which progressed to somnolence within one hour. There was no history of fever, seizure, headache, or focal neurological deficit. He denied alcohol consumption, smoking, or drug use. His past medical history included mild hypothyroidism under stable treatment with levothyroxine 25 µg daily. On admission, the patient was conscious but slow to respond. He was markedly lean, with a body mass index (BMI) of 14.4 kg/m^2^. Vital signs were stable. There was no jaundice, hepatosplenomegaly, or stigmata of chronic liver disease. Neurological examination was normal, without asterixis or focal deficits.

Baseline clinical and biochemical characteristics are summarized in Table 1, and additional diagnostic, imaging, electrophysiological, and genetic findings are presented in Table 2. Laboratory evaluation revealed mild elevation of AST (43 U/L) and ALT (52 U/L), with slightly increased bilirubin and marked hyperammonemia (103.07 µmol/L). Serum ceruloplasmin was decreased, but no Kayser–Fleischer rings were observed. Other tests, including viral and autoimmune markers, were unremarkable. Brain MRI and EEG were normal, whereas abdominal CT showed no hepatic abnormalities or portosystemic shunts (Figure 1B and Figure 2B, respectively). The patient was diagnosed with hyperammonemic encephalopathy and treated with lactulose, rifaximin, and LOLA, leading to full recovery within one day and normalization of plasma ammonia to 21 µmol/L after two days. Given his young age, recurrent episodes, and absence of cirrhosis or shunt, a urea-cycle or related metabolic disorder was suspected. Genetic testing revealed a homozygous c.852_855del (p.Met285ProfsTer2) mutation in the *SLC25A13* gene, confirming AACD.

Long before the onset of neurological symptoms, he had consistently preferred protein- and fat-rich foods and avoided sweets, rice, and other carbohydrate-rich foods. He was placed on a low-carbohydrate, protein- and fat-enriched diet supplemented with MCT, after which plasma ammonia remained normal and no further encephalopathic episodes occurred during follow-up.

### 2.3. Genetic Analysis

For both patients, genomic DNA was extracted from peripheral blood leukocytes and analyzed by whole-exome sequencing. Sequence alignment and variant calling were performed using the human reference genome GRCh38. Variants were interpreted according to the American College of Medical Genetics and Genomics/Association for Molecular Pathology guidelines. Both patients harbored the homozygous pathogenic *SLC25A13* variant NM_014251.3:c.852_855del (p.Met285ProfsTer2), which was confirmed by Sanger sequencing. Variant nomenclature followed the Human Genome Variation Society recommendations.

Plasma amino acid and acylcarnitine profiling were not available during the acute admissions. Therefore, plasma citrulline, arginine, glutamine, the threonine-to-serine ratio, and acylcarnitine species were not measured. The absence of these biochemical profiles limited the metabolic characterization of the two patients. Nevertheless, the diagnosis was established based on the compatible clinical phenotype, recurrent hyperammonemia, exclusion of relevant acquired causes, and identification of a homozygous pathogenic *SLC25A13* variant in both patients.

Apart from the maternal uncle described in Case 1, no additional family member was known to have recurrent encephalopathy, unexplained hyperammonemia, neonatal cholestasis, or similar dietary preferences. Familial segregation analysis was not performed during the current evaluation because relatives were not available for molecular testing. Genetic counseling and targeted testing for the familial *SLC25A13* variant were recommended for the parents, siblings, and other clinically relevant relatives. Such testing may identify heterozygous carriers and potentially unrecognized affected family members and will be pursued if the relatives provide consent and testing becomes feasible.

In both patients, hyperammonemia was documented during symptomatic exacerbations and decreased in parallel with neurological improvement after ammonia-lowering therapy. Available follow-up measurements suggested an episodic rather than continuously severe pattern. During 6 months of follow-up after discharge, plasma ammonia remained within the normal range, and no further encephalopathic episodes occurred.

## 3. Discussion

Citrin deficiency is a rare autosomal recessive metabolic disorder that can lead to adult-onset encephalopathy. It results from mutations in *SLC25A13* [4], which encodes the mitochondrial aspartate–glutamate carrier involved in the malate–aspartate NADH shuttle. Loss of citrin function impairs the exchange of metabolites between mitochondria and cytosol, reducing hepatic energy production and limiting aspartate availability for the urea cycle. As a result, ammonia and citrulline accumulate, disrupting nitrogen metabolism and leading to hyperammonaemia [5,6]. This disorder is most prevalent in East Asia, particularly in Japan, where the carrier frequency is approximately 1 in 42 and the estimated prevalence is around 1 in 7000 individuals [3]. AACD represents the late-onset phenotype of citrin deficiency and corresponds to the condition previously known as CTLN2 [9]. Patients are usually lean with low BMI and often prefer protein- and fat-rich foods while avoiding carbohydrates and alcohol. Episodes are commonly triggered by alcohol, high-carbohydrate intake, medication, or surgery, and many develop postprandial hyperammonaemia after evening meals, presenting with confusion or behavioral disturbance at night [9,10].

Plasma amino acid analysis can provide an important biochemical clue to AACD. Typical abnormalities include elevated citrulline and arginine concentrations and an increased threonine-to-serine ratio [9,10]. However, these findings may vary with metabolic status and are not independently sufficient to characterize the underlying *SLC25A13* genotype. In our patients, amino acid profiling was not performed because it was unavailable during the acute admissions. This represents a limitation of the present report, although the diagnosis was molecularly confirmed in both cases.

Clinical features include episodes of hyperammonaemia with variable neuropsychiatric and neurological symptoms, ranging from agitation and nocturnal delirium to disorientation, tremor, seizures, or coma [9,11]. Symptoms often fluctuate with ammonia levels, and misdiagnosis as hepatic encephalopathy or psychiatric disease is frequent, as liver function is typically preserved or only mildly impaired. Some patients later develop complications such as hyperlipidaemia, pancreatitis, or hepatocellular carcinoma [9]. Diagnosis relies on the detection of hyperammonaemia and confirmation of *SLC25A13* mutations. Early recognition is essential, as timely dietary management and ammonia-lowering therapy can reduce recurrence, whereas liver transplantation remains the only definitive treatment [9,10].

Our two cases highlight the variable clinical expression of AACD. Both patients were young adult males, consistent with the known male predominance of this disorder, and presented with repeated episodes of confusion and abnormal behavior associated with marked hyperammonaemia. Their lean body build and long-standing preference for protein- and fat-rich foods, together with avoidance of carbohydrates, were characteristic of citrin deficiency.

The first patient (21 years old) had recurrent nocturnal episodes of agitation and confusion with mild cirrhosis. However, the degree of liver damage was insufficient to explain the repeated episodes of encephalopathy, suggesting that the primary cause was metabolic rather than structural. The nocturnal onset of symptoms was consistent with previous reports that ammonia levels in AACD tend to rise after evening meals. This patient also had a history of neonatal jaundice, suggesting an earlier phase of citrin deficiency, and a maternal uncle with similar adult-onset behavioral disturbances previously misdiagnosed as epilepsy, further supporting the hereditary nature of the disease.

The history of neonatal jaundice in Case 1 raises the possibility of previously unrecognized neonatal intrahepatic cholestasis caused by citrin deficiency. Although the overt cholestatic manifestations of NICCD usually improve during infancy, citrin deficiency represents an age-dependent disease spectrum, and some patients may subsequently develop persistent metabolic liver abnormalities, steatosis, steatohepatitis, fibrosis, or cirrhosis. Therefore, the mild cirrhosis observed in Case 1 may represent long-term hepatic involvement associated with citrin deficiency. However, neonatal medical records, longitudinal liver assessments, and liver histology were unavailable; therefore, a direct progression from neonatal cholestasis to cirrhosis cannot be established in this patient.

In contrast, the second patient (18 years old) had transient, self-limited episodes of encephalopathy with only mild biochemical liver abnormalities and no structural changes on imaging, representing the milder end of the AACD spectrum.

Comprehensive evaluation in both patients excluded viral, autoimmune, and Wilson’s disease, and no evidence of portosystemic shunting was found. EEG in Case 1 showed diffuse slow-wave activity compatible with metabolic encephalopathy. The small occipital enhancing lesion and subtle meningeal enhancement were nonspecific and, given the normal cerebrospinal fluid findings and rapid clinical resolution without lesion-directed treatment, were not considered responsible for the recurrent neuropsychiatric episodes. Brain MRI and EEG were normal in Case 2. The persistence of hyperammonaemia despite relatively preserved liver function led to suspicion of a urea cycle–related metabolic defect, and genetic testing confirmed homozygous *SLC25A13* mutations (c.852_855del; p.Met285ProfsTer2) in both patients, establishing the diagnosis of AACD.

The *SLC25A13* c.852_855del (p.Met285ProfsTer2) variant is a recurrent pathogenic loss-of-function variant that has also been reported in earlier literature as 851del4 or c.851_854del. It is particularly frequent among individuals of East and Southeast Asian ancestry [7,8]. In a Vietnamese pediatric NICCD cohort, the corresponding deletion accounted for 91.78% of identified mutant alleles [8]. However, a robust genotype–phenotype correlation has not been established. Individuals carrying the same biallelic variant may differ in age at onset, severity of hyperammonemia, hepatic involvement, and progression across the citrin-deficiency spectrum. The markedly different hepatic and neuropsychiatric presentations of our two patients, despite an identical homozygous variant, further illustrate this phenotypic variability. Nevertheless, two cases are insufficient to establish a specific genotype–phenotype association, and environmental, nutritional, and additional genetic modifiers should be considered [11].

The difference in presentation between these two cases may reflect variations in hepatic metabolic reserve and exposure to triggering factors such as large meals, fatigue, or carbohydrate intake. Both patients responded well to ammonia-lowering therapy and a low-carbohydrate, protein- and fat-enriched diet supplemented with MCT, achieving sustained normalization of plasma ammonia and remaining symptom-free throughout the 6-month follow-up period.

The contribution of these cases does not lie in the identification of a novel genetic variant or an entirely new clinical manifestation. Rather, the report provides several clinically relevant diagnostic lessons. First, it documents two genetically confirmed Vietnamese young adults presenting with recurrent hyperammonemic neuropsychiatric episodes, a phenotype that remains sparsely described in the Vietnamese adult literature. Second, the two patients carried the same homozygous pathogenic variant but showed markedly different hepatic phenotypes: Case 1 had a history suggestive of neonatal citrin deficiency, mild cirrhosis, and severe behavioral episodes, whereas Case 2 had no structural liver disease and experienced shorter, self-limited episodes. Third, the cases illustrate how AACD may be mistaken for epilepsy, intracranial disease, Wilson’s disease, primary psychiatric illness, or conventional hepatic encephalopathy. Finally, they emphasize the diagnostic value of recognizing longstanding food preferences and hyperammonemia that is disproportionate to the severity of liver disease.

Nutritional management in citrin deficiency differs from the conventional high-carbohydrate, protein-restricted approach sometimes used for other hyperammonemic disorders. Patients should receive adequate total energy, avoid prolonged fasting, and limit excessive carbohydrate intake, particularly refined sugars and large carbohydrate loads. Published nutritional recommendations suggest an approximate protein-fat-carbohydrate energy distribution of 15–25%, 40–50%, and 30–40%, respectively, although the prescription should be individualized according to nutritional status, hepatic function, lipid profile, and metabolic response [10]. Adequate protein intake and relatively higher fat intake should be maintained, and MCT supplementation may be divided among meals. Alcohol and high-glucose or fructose-containing intravenous solutions should be avoided because they may precipitate metabolic deterioration [9,10].

This report has several limitations. Plasma amino acid and acylcarnitine profiling were unavailable, and serial ammonia measurements were not systematically obtained during untreated asymptomatic periods. In addition, no follow-up brain MRI was available for Case 1, and familial segregation analysis was not performed. Molecular testing of parents, siblings, and other clinically relevant relatives would confirm variant segregation and carrier status and could identify asymptomatic or minimally symptomatic individuals who may benefit from genetic counseling, dietary guidance, and clinical surveillance.

## 4. Conclusions

These cases illustrate the clinical heterogeneity of AACD and emphasize the need to consider citrin deficiency in adolescents and adults presenting with recurrent encephalopathy and otherwise unexplained hyperammonemia, particularly when ammonia levels are disproportionate to the severity of liver disease. Longstanding preference for protein- and fat-rich foods and avoidance of carbohydrate-rich foods may provide an important diagnostic clue. Although plasma amino acid analysis can support the diagnosis, molecular confirmation of biallelic pathogenic *SLC25A13* variants is essential. Early recognition, appropriate nutritional management, and genetic counseling may prevent recurrent metabolic decompensation and neurological injury.

## Figures and Tables

**Table 1:** Baseline clinical and biochemical characteristics of the two patients with AACD on admission.

Patients	Case 1	Case 2	Reference Range
Age (years)	21	18	
Sex	Male	Male	
BMI (kg/m^2^)	17.2	14.4	
Dietary preference	High protein, avoids rice and sweets	High protein and fat, low carbohydrate	
AST (U/L)	43	43	<40
ALT (U/L)	84	52	<41
GGT (U/L)	553	85	<40
ALP (U/L)	NA	106.53	43–115
Total Bilirubin (mg/dL)	0.93	2.21	<1.02
Albumin (g/L)	42.97	38.9	35–52
Urea (mg/dL)	26.36	25.17	10.2–49.7
Creatinine (mg/dL)	1.09	0.82	0.83–1.28
Sodium (mmol/L)	142	141	136–146
Potassium (mmol/L)	3.17	3.42	3.4–5.1
INR	1.22	1.22	0.8–1.2
Ammonia (µmol/L)	345.21	103.07	16–60
White blood cell (G/L)	7.26	9.16	4–10
Hemoglobin (g/L)	160	142	120–175
Platelet (G/L)	117	185	150–450

NA, not available. The serum alkaline phosphatase value for Case 1 was not available in the original medical record.

**Table 2:** Additional biochemical, diagnostic, imaging, electrophysiological, and genetic findings in the two patients with AACD.

Findings	Case 1	Case 2
Viral markers (HBV, HCV, CMV, EBV, HSV)	Negative	Negative
Autoimmune markers	Negative	Negative
Ceruloplasmin	0.90	0.95
Kayser–Fleischer rings	Absent	Absent
24-h urinary copper	13 µg/day	16 µg/day
Cerebrospinal fluid analysis	Normal	Not performed
EEG	Diffuse slow-wave activity without epileptiform discharges	Normal
Brain MRI	Small nonspecific enhancing lesion beneath the left occipital cortex with subtle meningeal enhancement; low-lying cerebellar tonsils approximately 7 mm below the foramen magnum	Normal
Abdominal CT	Compensated cirrhotic morphology with recanalization of the umbilical vein; no portosystemic shunt	Normal liver; no portosystemic shunt
Plasma amino acid profile	Not performed	Not performed
Acylcarnitine profile	Not performed	Not performed
*SLC25A13* variant	NM_014251.3:c.852_855del (p.Met285ProfsTer2), homozygous	NM_014251.3:c.852_855del (p.Met285ProfsTer2), homozygous

AACD, adolescent and adult citrin deficiency; CT, computed tomography; EEG, electroencephalography; MRI, magnetic resonance imaging.

## Data Availability

The data presented in this report are available from the corresponding author upon reasonable request. The data are not publicly available because of patient privacy and ethical restrictions.

## Abbreviations

ALP	alkaline phosphatase
ALT	alanine aminotransferase
AST	aspartate aminotransferase
BMI	body mass index
CMV	cytomegalovirus
CT	computed tomography
CTLN2	adult-onset citrullinaemia type II
CSF	cerebrospinal fluid
EBV	Epstein–Barr virus
EEG	electroencephalography
GGT	γ-glutamyl transferase
INR	international normalized ratio
LOLA	L-ornithine–L-aspartate
MCT	medium-chain triglycerides
MRI	magnetic resonance imaging
NADH	nicotinamide adenine dinucleotide (reduced form)
